# Recapitulating and repairing STAT1 gain-of-function using patient-derived expanded potential stem cells (EPSC): a proof-of-concept of EPSC to study inborn errors of immunity

**DOI:** 10.1016/j.jaci.2023.11.914

**Published:** 2023-12-09

**Authors:** Xueyan Liu, Vera SF Chan, Kenneth GC Smith, Chang Ming, Chung Sze Or, Faria TW Tsui, Bo Gao, Matthew C Cook, Pentao Liu, Chak Sing Lau, Philip Hei Li

**Affiliations:** 1 Centre for Translational Stem Cell Biology, University of Hong Kong, Hong Kong; 2 Division of Rheumatology and Clinical Immunology, Department of Medicine, Queen Mary Hospital, University of Hong Kong, Hong Kong; 3 Department of Medicine, University of Cambridge School of Clinical Medicine, University of Cambridge, Cambridge, United Kingdom; 4 School of Biomedical Sciences, University of Hong Kong, Hong Kong; 5 School of Biomedical Sciences, Chinese University of Hong Kong, Hong Kong

**Keywords:** Expanded Potential, Gain-of-Function, Gene editing, Immunodeficiency, Inborn errors of immunity, JAK inhibitor, Model, Personalized, STAT1, Stem cell, CRISPR/Cas9, expanded potential stem cell, Janus-kinase inhibitor, gain of function, phosphorylation, signal transducer and activator of transcription 1

## Abstract

**Background:**

Inborn errors of immunity (IEI) often lack specific disease models and personalized management. Signal transducer and activator of transcription (STAT)-1 gain-of-function (GoF) is such example of an IEI with diverse clinical phenotype with unclear pathomechanisms and unpredictable response to therapy. Limitations in obtaining fresh samples for functional testing and research further highlights the need for patient-specific *ex-vivo* platforms.

**Objective:**

Using STAT1-GoF as an example IEI, we investigated the potential of patient-derived expanded potential stem cells (EPSC) as an *ex-vivo* platform for disease modelling and personalized treatment.

**Methods:**

We generated EPSC derived from individual STAT1-GoF patients. *STAT1* mutations were confirmed with Sanger sequencing. Functional testing including STAT1 phosphorylation/dephosphorylation and gene expression with or without Janus-kinase inhibitors (JAKi) were performed. Functional tests were repeated on EPSC lines with GoF mutations repaired by CRISPR/Cas9 editing.

**Results:**

EPSC were successfully reprogrammed from STAT1-GoF patients and expressed the same pluripotent makers as controls, with distinct morphological differences. Patient-derived EPSC recapitulated the functional abnormalities of index STAT1-GoF patients with STAT1 hyperphosphorylation and increased expression of *STAT1* and its downstream genes (*IRF1, APOL6*, and *OAS1)* following IFNγ stimulation. Addition of ruxolitinib and baricitinib inhibited STAT1 hyperactivation in STAT1-GoF EPSC in a dose-dependent manner, which was not observed with tofacitinib. Corrected STAT1 phosphorylation and downstream gene expression were observed among repaired STAT1-GoF EPSC cell lines.

**Conclusion:**

This proof-of-concept study demonstrates the potential of our patient-derived EPSC platform toward modelling STAT1-GoF. We propose this platform toward researching, recapitulating and repairing other IEI in the future.

## Introduction

Inborn errors of immunity (IEI) often lack specific disease models and personalized management. Signal transducer and activator of transcription (STAT)-1 gain-of-function (GoF) is such example of an IEI with diverse clinical phenotype with unclear pathomechanisms and unpredictable response to therapy ^1–3^. First described in 2011 as an “autosomal dominant chronic mucocutaneous candidiasis (CMC) disease”, the spectrum of STAT1-GoF manifestations and severity varies greatly even among family members harboring the same germline mutation. Some patients can develop relatively mild or late-onset CMC while others can develop life-threatening infections, autoimmunity or even malignancy ^4, 5^. This heterogeneity may also be contributed by undiscovered regulatory elements or an interplay with variants of other loci, a phenomenon now recognized in other IEI ^6^.

Given the rarity of IEI, much of the underlying pathophysiology and mechanisms of individual IEI such as STAT1-GoF remain unclear. Alike its diverse clinical phenotype, the underlying mechanisms of STAT1-GoF and STAT1 hyperactivation may also differ between mutations ^7–9^. Furthermore, studying or confirming the function of individual mutations remain difficult, especially given the limited availability of fresh patient samples. Patients with IEI inherently suffer from frequent infections or are already receiving immunosuppressants even at the time of initial diagnosis, making them unavailable or unsuitable for frequent blood sampling.

The conventional management of many IEI, including STAT1-GoF, includes the use of long-term antimicrobial prophylaxis and immunosuppression for autoimmune manifestations. However, use of long-term anti-microbial is associated with development of multi-drug resistance and immunosuppressive therapies may further exacerbate susceptibility to infections or malignancy. Although potentially curative, the outcomes of hematopoietic stem cell transplantation (HSCT) for STAT1-GOF have been poor, associated with high rates of graft failure and low overall survival ^10^. More recently, the use of Janus Activating Kinase (JAK) inhibitors (JAKi) has shown promise and demonstrated effectiveness in ameliorating both *Candida* susceptibility and autoimmunity among STAT1-GoF patients. A variety of JAKi with different selectivity (such as JAK1/2-selective ruxolitinib and baricitinib, JAK1/3-selective tofacitinib or JAK1-selective itactinib), have been reported with variable success ^11–17^. Unfortunately, there have also been reports of failed JAKi with subsequent worsening of fungal and viral infections ^16, 18^. Given the complexity of STAT1-GoF pathophysiology and patient heterogeneity, individual patients will likely respond differently to discrete JAKi depending on their underlying disease mechanisms. This highlights the need for *ex-vivo* and patient-specific testing platforms, which would enable evaluation of novel therapies irrespective of what current treatments the patients are already receiving.

Although various models such as mice or leukemia-derived cell lines with artificially induced STAT1-GoF mutations have been reported, none have yet to recapitulate mutation or patient-specific properties, and in the context of the individual patient’s genome ^19, 20^. Especially given the poor genotype-phenotype correlation in STAT1-GoF, disease models using artificially induced mutations would miss effects from regulatory or modifier genes beyond *STAT1* and lack individual specificity. Adapting from established protocols for human expanded potential stem cells (EPSC), our group generated EPSC cell lines by reprogramming erythroid progenitor cells (EPC) derived from two patients with confirmed STAT1-GoF mutations of varying clinical severity ^21^. Uniquely, EPSC are a type of totipotent stem cell which can be established by reprogramming somatic cells and possess superior developmental potency for all cell lineages ^21^. For example, EPSC demonstrate superior directed differentiation potential to generate functional hepatocytes transcriptionally closer to the primary human hepatocytes compared with embryonic stem cells ^22, 23^. They also have a higher proliferation rate and better genetic and epigenetic stability, making them a more suitable platform for human disease modelling and gene editing ^24^. Compared to EPSC derived from healthy controls, we confirmed intact *STAT1* GoF mutations and STAT1 hyperactivation from patient derived EPSC. Furthermore, using CRISPR/Cas9 editing, we were able to repair the GoF mutation in patient-derived EPSC lines which demonstrated functional correction of STAT1 phosphorylation and downstream gene expression.

## Methods

### Patient selection

Peripheral blood mononuclear cells (PBMC) were isolated from peripheral blood from STAT1-GoF patients and healthy controls for processing. All blood donors gave informed consent and the study was approved by the Institutional Review Board of the University of Hong Kong/Hospital Authority Hong Kong West Cluster.

### EPSC reprogramming, maintenance and characterization

EPSC reprogramming was performed from patient or control PBMC-derived EPC by CytoTune iPS 2.0 Sendai Reprogramming Kit (Thermo Fisher Scientific) according to the manufacturer’s protocol with slight modifications. Briefly, 1×10^6^ PBMC were cultured in EPC expansion medium containing StemSpan™ SFEM II (STEMCELL Technologies) basal medium and 1x StemSpan™ Erythroid Expansion Supplement (STEMCELL Technologies), with medium changed every 2 days. Day 9 EPC-expanded culture with >90% cells expressing EPC maker CD71 was used for EPSC reprogramming. 3×10^5^ EPC were transduced by spinoculation with three Sendai viruses (5 MOI each) that contained polycistronic Klf4–Oct3/4–Sox2, cMyc, and Klf4. Transduced EPC was then cultured in EPC expansion medium in a 48-well plate for 24 hours before removal of viruses in culture medium. Cells were cultured in EPC expansion medium for 2 more days, and then transferred to a 6-well feeder plate coated with irradiated mouse embryonic fibroblasts. The culture continued in StemSpan™ SFEM II medium for another 6 days with fresh medium change every other day. On day-9 post-transduction, half of the culture medium was replaced with EPSC culture medium (EPSCM: 10mM CHIR99021, 50mM XAV939, 50mM Endo-IWR-1, and 3mM A419259) and cultured for another day. The culture then continued with daily full change of EPSCM. EPSC colonies were picked from day-17 to day-20 for maintenance and characterization.

To maintain EPSC, the picked single EPSC colony was digested into single cells by TrypLE™ Express Enzyme (Gibco™), then seeded in a 48-well feeder plate and cultured in EPSCM for 5 days. EPSC was then gradually passaged to a 24-well and then a 12-well feeder plate for expansion and maintenance. A feeder system was used to allow EPSC to be maintained for long-term culture. Single-colony picking for subcloning was performed 3-4 times to derive EPSC clones from each individual. Disappearance of viral vector was verified by PCR using recommended primer sets of the Sendai Reprogramming Kit. To verify the genotype of EPSC from each individual, the DNA binding domain on STAT1 cDNA was amplified by PCR and followed by Sanger sequencing. To characterize the pluripotency of reprogrammed EPSC, the expression of pluripotent markers was checked by quantitative real-time PCR (qRT-PCR) and immunofluorescence staining.

### RNA isolation and quantitative real-time PCR

RNA analysis was performed to determine gene expression levels under various conditions. Experiments were repeated at least two times with technical triplicate for each gene. To determine gene expression in EPSC, cells were grown in EPSCM with or without IFNγ (STEMCELL Technologies 78020.1) stimulation or the addition of JAKi and harvested at indicated time points (normally reaching 70–80% confluence in a 24- or 12-well plate). Cellular RNA was extracted using Trizol (Invitrogen) according to the recommended procedure. 1ug of RNA was reverse-transcribed using PrimeScript™ RT Master Mix (Takara) following the manufacturer’s protocol. qRT-PCR using PowerUp™ SYBR™ Green Master Mix (Thermo Fisher Scientific) was performed on an QuantStudio™ 7 Flex Real-Time PCR System, 384-well, desktop (Applied Biosystems™). Quantification of pluripotent genes (*NANOG, OCT4, SOX2, REX1* and *SALL4)*, IFN receptor genes *(IFNGR1, IFNGR2, IFNAR1* and *IFNAR2*), *STAT1* and *STAT1* downstream genes (*IRF1, APOL6, OAS1* etc.) was performed using primers listed in Table E1. Target gene expression was normalized to the expression of the housekeeping gene *GAPDH*. The relative expression levels were compared using the 2^-ΔCt^ method.

### Immunofluorescence staining

EPSC pluripotent markers expression and level of phosphorylated STAT1 (p-STAT1) following IFNγ stimulation were determined by immunofluorescence staining. 70-80% confluent EPSC cultures were fixed with 4% formaldehyde (Servicebio) for 10 minutes, permeabilized by 0.2% Triton-X 100 (Sigma-Aldrich) in PBS for 10 minutes, washed with PBST and blocked for an hour in blocking solution (Servicebio). Cells were then incubated in blocking buffer with primary antibodies at 1:200 (Rabbit anti-Mouse/Human Oct4, StemAb™ 09-0023; Rabbit anti-Mouse/Human Nanog, StemAb™ 09-0020; Alexa Fluor™ 488 conjugated TRA-1-60, Invitrogen™ A25618) overnight at 4 °C. After washing, cells were stained with a secondary antibody (Alexa Fluor™ 594 conjugated donkey anti-Rabbit IgG, Invitrogen A32754) at 1:1000 for 1 hour at room temperature. Cells were then thoroughly washed and imaged using Ts2-FL Compact Inverted Microscope for Fluorescence (Nikon).

For p-STAT1 expression assays following IFNγ stimulation, EPSC were seeded in a 24-well feeder plate at 2×10^5^ */* well and cultured for 4 days. Cells were then stimulated for 1 hour with 50ng/ml IFNγ, followed by fixation for 10 minutes. Cells were then permeabilized using ice-cold methanol and kept at -20 °C for 30 minutes, followed by washing and blocking as above. Staining was performed using rabbit anti-p-STAT1 (Cell signaling Technology 7649S) primary antibody and the above-mentioned secondary antibody. Image was taken by PE Spinning Disc Ultraview or Ts2-FL Compact Inverted Microscope for Fluorescence. Acquired images were analyzed using ImageJ (NIH, Version 2.1.0/1.53c) The mean fluorescence intensity from more than 10 nuclei was quantified for each group.

### Immunoblotting

Immunoblotting was used to determine p-STAT1 and total STAT1 (t-STAT1) protein levels. To examine p-STAT1 levels of EPSC following stimulation, 70-80% confluent EPSC were stimulated with or without 50ng/ml IFNγ for 1 hour and harvested. To determine STAT1 dephosphorylation, EPSC was first stimulated with 50ng/ml IFNγ for 1 hour, then immediately washed with PBS, and further incubated with fresh medium (for IFNγ withdrawal) or fresh medium containing 1uM ruxolitinib (Selleck S1378) and harvested at indicated time points. To compare responses between different JAKi on p-STAT1 inhibition during IFNγ stimulation, EPSC were stimulated with 50ng/ml IFNγ with or without the presence of 1, 10, 100, or 1000 nM of JAKi [baricitinib (MedChemExpress, HY-15315), ruxolitinib, and tofacitinib (MedChemExpress, HY-40354)] for 24 hours. Untreated EPSC was used as a control.

For immunoblotting analysis, cells were lysed in RIPA Lysis and Extraction Buffer (Thermo Scientific™) supplemented with 1x Halt™ Protease and Phosphatase Inhibitor Cocktail (Thermo Fisher Scientific) for 30 minutes on ice. Lysates were then centrifuged at 12,000 x *g* for 10 minutes at 4 °C. Protein concentrations were determined using the Pierce™ BCA Protein Assay Kit (Thermo Fisher Scientific). Samples were then prepared by the addition of NuPAGE™ LDS Sample Buffer (4x) (Thermo Fisher Scientific) followed by boiling at 95 °C for 5 minutes. Samples were subjected to SDS-PAGE separation by running 10μg protein on a 15-well 10% Bis-Tri gel (Invitrogen™), transferred to a nitrocellulose membrane (Bio-Rad). Membranes were blocked in 5% milk (Bio-Rad) in TBST (Sigma-Aldrich) for 2 hours at room temperature, followed by incubation with rabbit anti-p-STAT1 (Cell signaling Technology, 7649S) or anti-β-actin antibodies (Cell signaling Technology, 4967S) at 4 °C overnight. Membranes were then washed and incubated for 1 hour at room temperature with HRP-conjugated goat anti-rabbit IgG secondary antibody (Invitrogen 31460). Membranes were imaged using Uvitec Alliance Q9 Advanced imaging system and analysed with ImageJ. Stat1 (D1K9Y) Rabbit mAb (Cell signaling Technology 14994) was used to blot t-STAT1 after stripping out the p-STAT1 primary antibody on the same membrane for each experiment.

### Flow cytometry

Flow cytometry was used to quantify p-STAT1 and t-STAT1 levels. To test the dynamic change of p-STAT1 in EPSC during IFNγ stimulation, EPSC were cultured in a 24-well plate as above and stimulated with 50ng/ml IFNγ. Cells were harvested at 0, 15, 30, and 60 minutes for intracellular staining. To test STAT1 dephosphorylation, EPSC were treated and harvested as in immunoblotting. To compare responses between different JAKi on p-STAT1 and t-STAT1 inhibition during IFNγ stimulation, EPSC or patient fresh blood leukocytes were seeded and stimulated with or without JAKi for 24 hours as in immunoblotting. To compare the p-STAT1 levels between original and repaired GoF EPSC following stimulation, EPSC were stimulated for 1 hour with 50ng/ml IFNγ. For flow cytometry analysis, cells were fixed using 4% FPA for 10 minutes and permeabilized using ice-cold methanol at -20 °C for 30 minutes. Cells were then washed 2 times using flow cytometry staining buffer (2% FBS in PBS) and stained using Alexa Fluor® 488 anti-Oct4 (Biolegend 653706) together with Alexa Fluor® 647 anti-STAT1 Phospho (Tyr701) (Biolegend 666410) or Alexa Fluor® 647 Mouse anti-Total Stat1 (BD Bioscience 558560) for 1 hour at 4 °C. Data was acquired with BD FACSymphony™A3 Cell Analyzer and analyzed using FlowJo™ v10. EPSC was identified as Oct4-positive cells. p-STAT1 and t-STAT1 quantification was measured as mean fluorescence intensity.

### CRISPR/Cas9 repairing of GoF mutation in STAT1-GoF EPSC

CRISPR/Cas9 editing was used to repair the pathogenic STAT1-GoF mutation in EPSC of patient 1 (P1). Briefly, EPSC were cultured to 80%~90% confluence and digested to single cells for electroporation using the Neon™ Transfection System. 2×10^5^ cells were used in each 10 μL Neon transfection reaction. 5 μg Cas9 (ThermoFisher A36499) and 1.5 μg sgRNA were gently mixed and left for complexing at room temperature for 15-20 minutes prior to cell electroporation. EPSC were resuspended in Resuspension Buffer R, gently mixed with 3.5 μg ssODN and left at room temperature for 3 minutes. The cell-ssODN suspension was then mixed with Cas9-sgRNA complex, and further incubated for 3-5 minutes. The electroporation was then done using a 10 μL Neon Tip on Neon™ Transfection System with pulse conditions of voltage: 1250 v, width: 20 ms, and pulse number: 2. Cells were immediately transferred to EPSCM with 10μM Y27632 (MedChemExpress HY-10583) in a 12-well feeder plate. Cells were then incubated at 37°C in a humidified CO_2_ incubator with daily full change of EPSCM. EPSC colonies emerged were picked and screened by genotyping. Sequences of ssRNA, ssODN and STAT1 genotyping primers were listed in Table E1.

### Statistical analysis

Statistical analysis was performed using GraphPad Prism 9 (version 9.3.1). The ordinary one-way ANOVA test or 2way ANOVA Dunnett’s multiple comparisons test was used to determine differences among groups. Statistical significance was represented as: *p < 0.05, **p < 0.01, ***p < 0.001, ****p < 0.0001.

## Results

### STAT1-GoF patient selection and clinical features

Two individual patients with confirmed STAT1-GoF mutations and variable clinical severity were selected. Patients were labeled as P1 and P2 and their clinical features are listed in Table 1. The specific mutations and loci of P1 and P2 are depicted in Figure E1.

### Defects in cell proliferation during EPC expansion among STAT1-GoF patients

**Figure 1:**
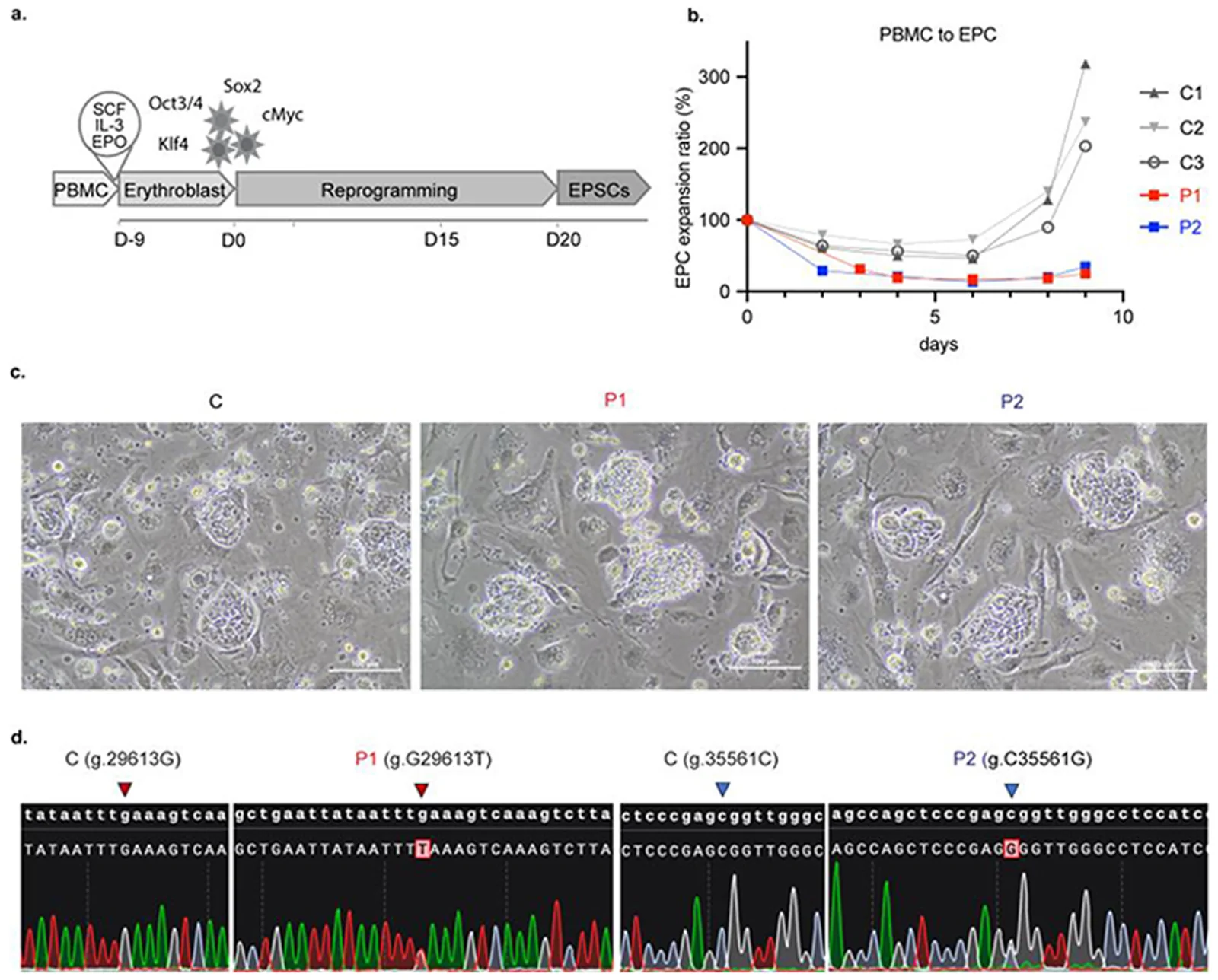
Generation of EPSC from STAT1-GoF patients. a) Flow chart of the reprogramming process of PBMC to EPSC; b) impaired cell proliferation during EPC expansion among patient-derived PBMC vs. controls; c) representative EPSC morphology during maintenance phase; d) Confirmation of intact point mutations from STAT1-GoF EPSC.

To avoid EPSC being reprogrammed from lymphocytes or other undesired cell types, PBMC from patients and controls were first expanded to EPC (Figure 1a). After 9 days of expansion, small lymphocytes were replaced by large, round, and middle brown cells (Figure E2a) with more than 90% expressing the EPC markers CD71 and/or CD235α by flow cytometry (Figure E2b). Expanded EPC downregulated gene expression of B lymphocyte (*CD19*), T lymphocyte (*CD3*), and monocyte (*CD14*) markers; and upregulated expression of *CD71, HBA1, HBB, and HBG1* (Figure E2c). In comparison to controls, patient derived PBMC demonstrated impaired cell proliferation during EPC expansion (Figure 1b).

### EPSC successfully reprogrammed from STAT1-GoF patients and controls

We used the Sendai programming kit to reprogram PBMC-derived EPC to EPSC. We observed successfully reprogrammed EPSC from day 12 and they were ready to be picked from day 15 to day 20 during reprogramming (Figure E3). All picked EPSC demonstrated self-renewal capabilities and could be maintained in EPSCM *in vitro* (Figure 1c). Single-colonies were picked to derive homogenous clones for each cell line. Viral vector clearance was confirmed by RT-PCR and qRT-PCR (Figure E4). To ensure that individual EPSC cell lines were derived from the correct patients and controls, sequence of the DNA binding domain of *STAT1* cDNA and gDNA for all EPSC cell lines was verified by Sanger sequencing. Patient-specific point mutations were confirmed for all patient-derived EPSC (Figure 1d), while control-derived EPSC demonstrated normal genotype.

### STAT1-GoF EPSC expressed same pluripotent makers as controls but were morphologically different

**Figure 2:**
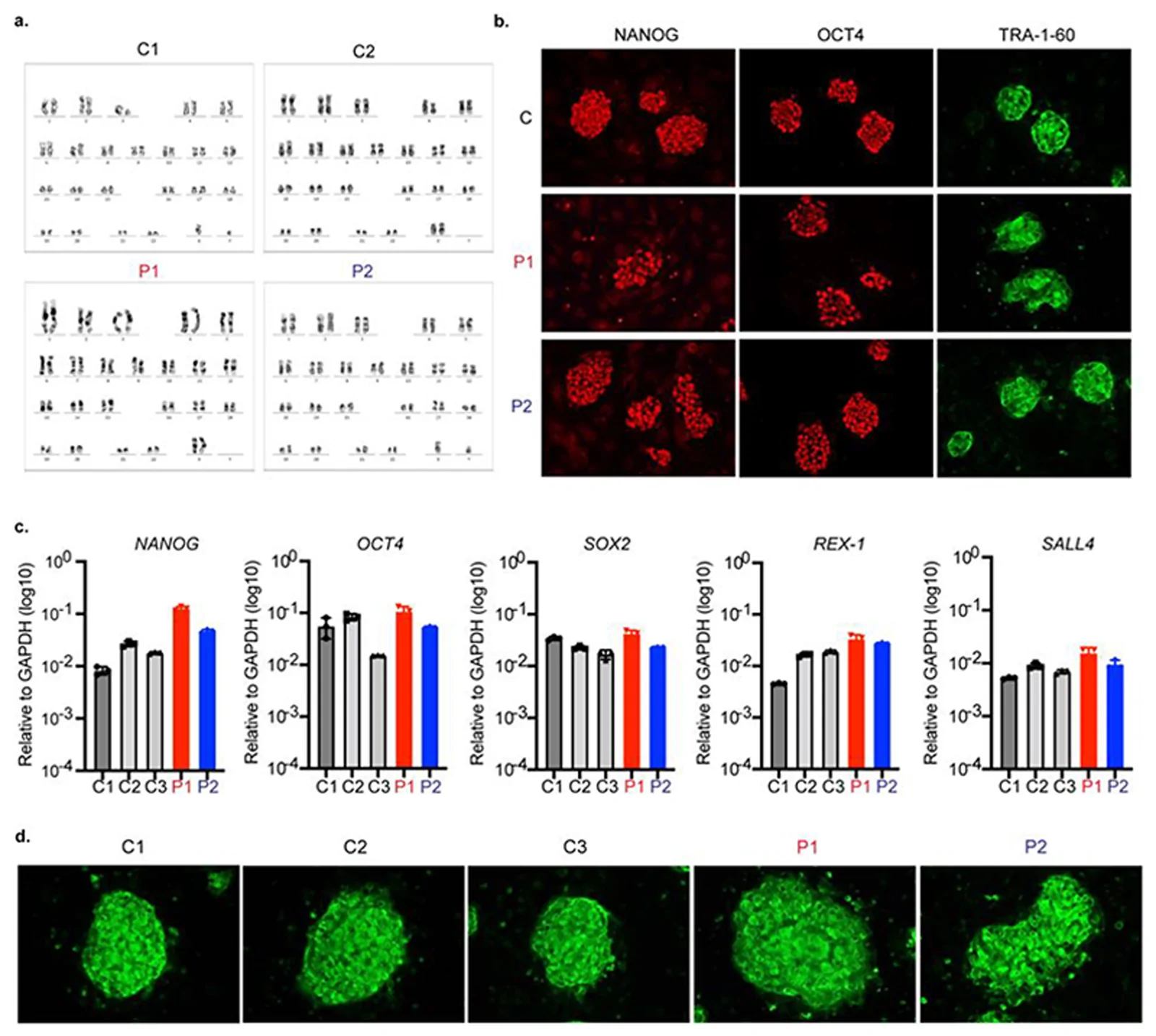
Characterization of pluripotency in STAT1-GoF and control EPSC. a) Normal karyotypes of STAT1-GoF and control EPSC; b) Comparable levels of NANOG, OCT4, and TRA-1-60 protein by immunofluorescence staining; c) Comparable expression of *NANOG, OCT4, SOX2, REX-1* and *SALL4*; d) STAT1-GoF EPSC were more flattened in morphology and exhibited rougher clone boundaries.

Patient and control EPSC demonstrated normal karyotypes (Figure 2a). EPSC pluripotency was confirmed by immunofluorescence staining of NANOG, OCT4, and TRA-1-60 protein expression (Figure 2b). Similarly, qRT-PCR demonstrated that STAT1-GoF EPSC expressed comparable levels of critical pluripotent marker genes (*NANOG, OCT4, SOX2, REX-1* and *SALL4*) compared to controls (Figure 2c). Although demonstrating comparable self-renewal capability and pluripotency with controls, STAT1-GoF EPSC were more flattened in morphology, exhibited rougher clone boundaries and were easier to be dissociated when compared to controls (Figure 2d and Figure E5).

### STAT1-GoF EPSC exhibited increased p-STAT1 following IFN stimulation

**Figure 3:**
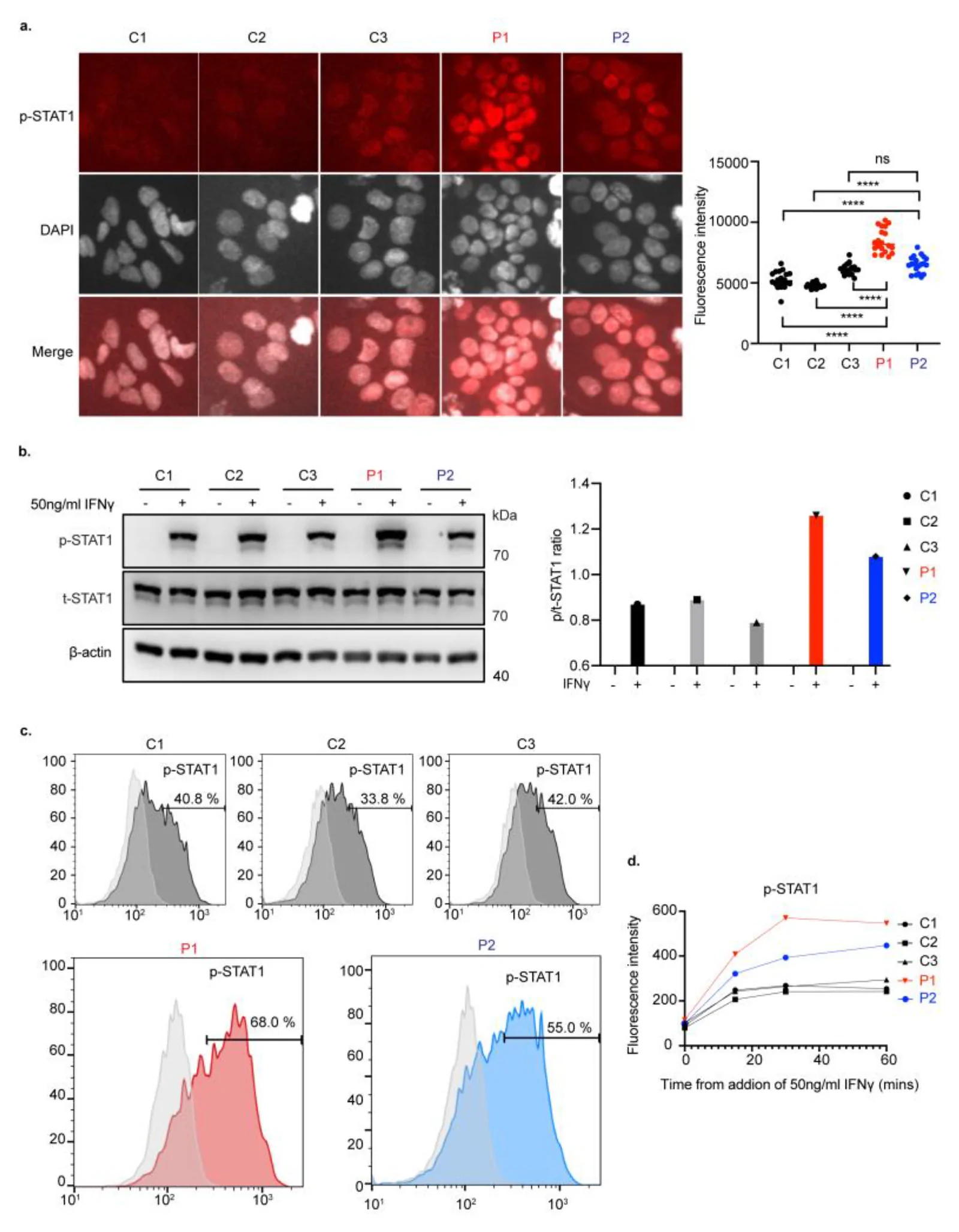
Increase in p-STAT1 of STAT1-GoF EPSC following short-term IFNγ stimulation. a) p-STAT1 detected by immunofluorescence (mean fluorescence intensity calculated from 20 nuclei of each sample); b) p-STAT1 measured by Western blot and relative intensity to t-STAT1; c) Proportion of EPSC expressing p-STAT1 at 0 and 15 minutes following stimulation; d) Mean fluorescence intensity of p-STAT1 among patient and control EPSC from 0 to 60 minutes following stimulation.

All EPSC expressed *STAT1*, type I (*IFNAR1* and *IFNAR2*), and type II (*IFNGR1* and *IFNGR2*) IFN receptor genes at relatively high levels in comparison to lineage markers (such as *GATA2*) at rest; suggesting potential response to IFN stimulation (Figure E6). Therefore, we stimulated EPSC with 50ng/ml of IFNγ for 1 hour which led to significant increase in p-STAT1 as detected by immunofluorescence (Figure 3a). Overall, quantification by immunoblotting demonstrated that STAT1-GoF EPSC expressed significantly higher p-STAT1 levels compared to controls (except the difference between P2 and C3 p-STAT1 did not reach statistical significance). Furthermore, similar findings were observed when p-STAT1 was detected by Western blot, with all STAT1-GoF demonstrating significantly higher increase in p/t-STAT1 ratios following IFN stimulation (Figure 3b).

The kinetics of p-STAT1 induction on EPSC were measured by flow cytometry at 0, 15, 30, and 60 minutes following IFNγ stimulation. To avoid unwanted signals from MEF cells (which were used as feeders for EPSC maintenance), only OCT4-positive cells were gated for quantification. At 15 minutes post-stimulation, 68.0% and 55.0% of EPSC derived from P1 and P2 expressed p-STAT1; respectively. This was significantly higher than the control EPSC (mean: 38.8%) (Figure 3c). Both the level and rate of STAT1 phosphorylation were greater among STAT1-GoF EPSC compared to controls among repeat experiments, with p-STAT1 levels around 3-fold higher for P1 EPSC and 1.5 to 2-fold higher for P2 EPSC when compared to control EPSC (Figure 3d).

### STAT1-GoF EPSC demonstrate delayed STAT1 dephosphorylation following addition of JAKi

**Figure 4:**
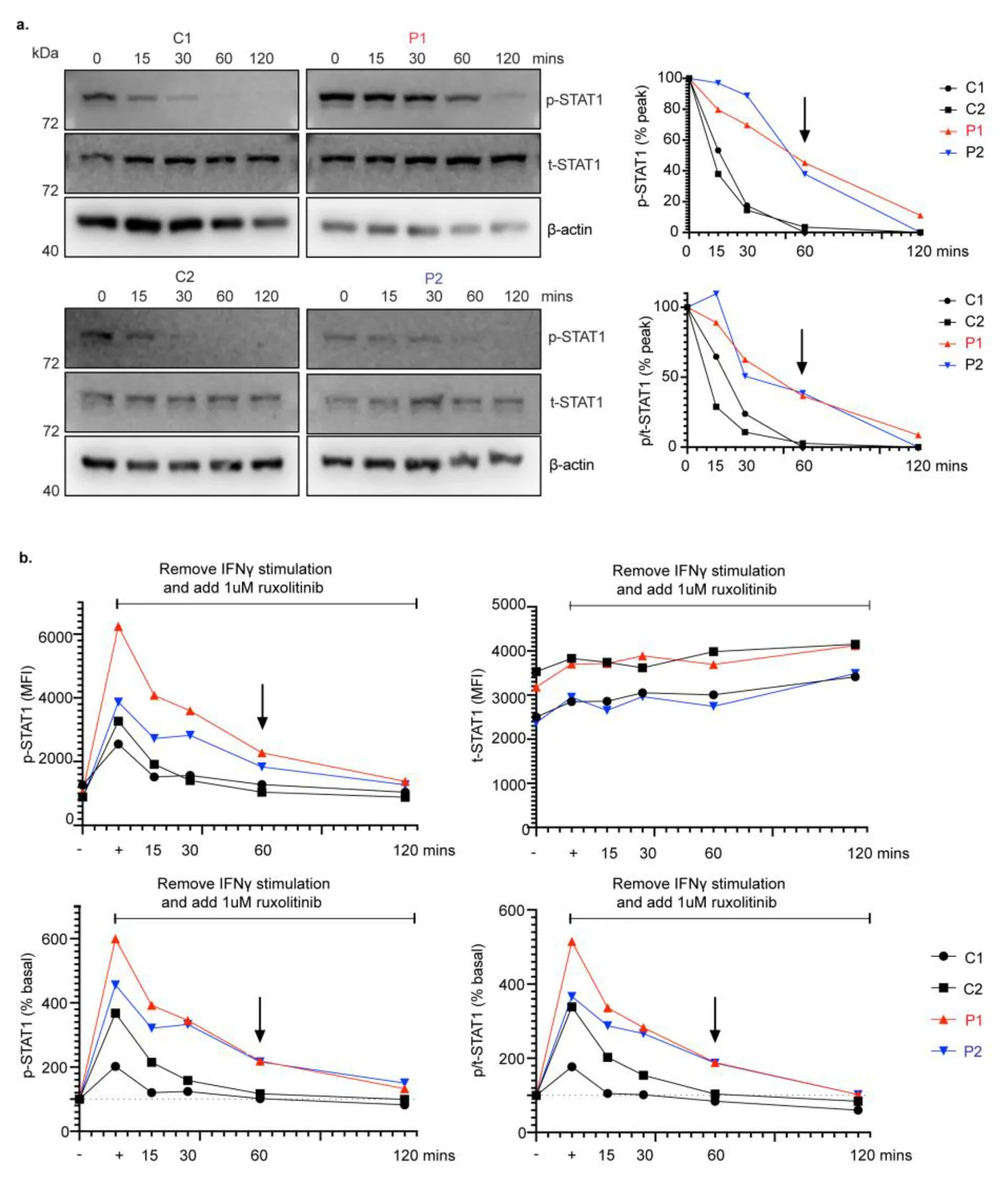
Delayed dephosphorylation of p-STAT1 in patient EPSC after addition of ruxolitinib following initial IFNγ stimulation by a) immunoblotting and b) flow cytometry. “-”: before IFN γ stimulation; “+”: 1 hour following stimulation with 50ng/ml IFNγ; “15”, “30”, “60” and “120”: minutes following addition of 1uM ruxolitinib.

For STAT1 dephosphorylation, p-STAT1 was measured after IFNγ stimulation following with either withdraw of IFNγ (by removal medium and washing with PBS then replaced by fresh medium) or addition of 1uM ruxolitinib. No obvious decrease of p-STAT1 was detected up to one and eight hours (Figure E7a), although there was increase in t-STAT1 detected following 2 and up to 8 hours following IFNγ withdraw (Figure E7b). In contrast, a progressive decrease in p-STAT1 was observed following addition of **1uM** ruxolitinib in all EPSC (Figure 4) within 2 hours. Compared to control EPSC, STAT1-GoF EPSC demonstrated a significantly delayed decrease in p-STAT1 (Figure 4).

### STAT1-GoF EPSC as a potential drug screening platform for specific JAKi

**Figure 5:**
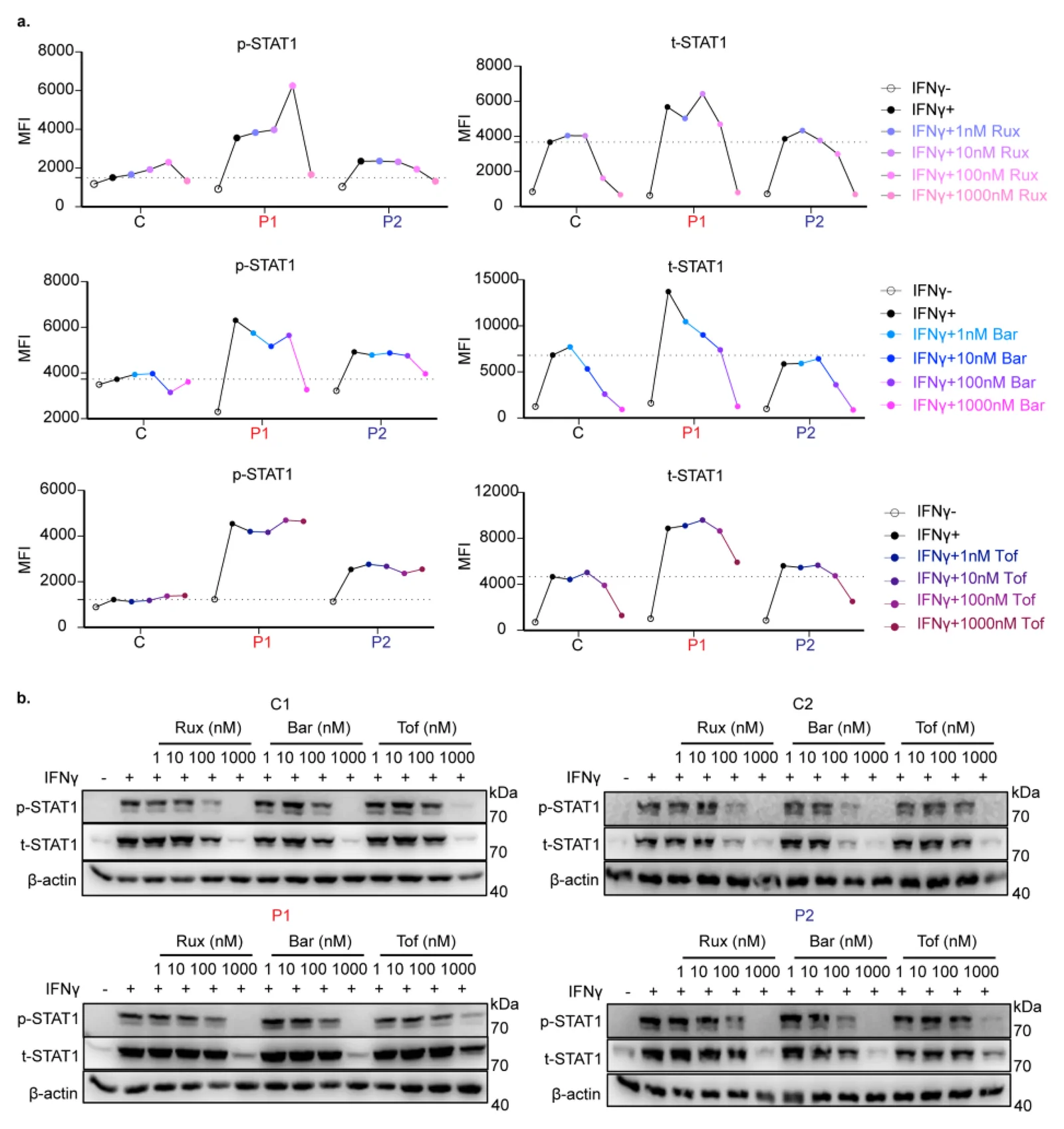
Effect of JAK inhibition on p-STAT1 and t-STAT1 expression. p-STAT1 and t-STAT1 levels among EPSC following 24-hour co-incubation with different concentrations of ruxolitinib (Rux), baricitinib (Bar) and tofacitinib (Tof) as measured by a) flow cytometry and b) Western blot.

To further investigate differential effects of specific JAKi during EPSC stimulation, JAKi were added at varying concentrations (1, 10, 100, and 1000 nM) together with 50ng/ml IFNγ into EPSC culture. p-STAT1 and t-STAT1 expression was then quantified by flow cytometry after 24-hour co-incubation. We noted differential patient-specific responses to different JAKi among different EPSC. Among STAT1-GoF EPSC, prolonged co-incubation with ruxolitinib and baricitinib resulted in significantly lower p-STAT1 at higher concentrations. However, there was no significant change in p-STAT1 following co-incubation with tofacitinib (Figure 5a). Concurrently, there was reduction of t-STAT1 expression following incubation of all three JAKi among both control and STAT1-GoF EPSC. Similar findings were demonstrated when experiments were repeated with p-STAT1 and t-STAT1 expression measured by Western blot (Figure 5b). Viability of cells were confirmed in all experiments, and β-actin was also quantified for comparison. These findings were similarly found when co-incubating JAKi with primary blood leukocytes from patient PBMC (Figure E8).

**Figure 6:**
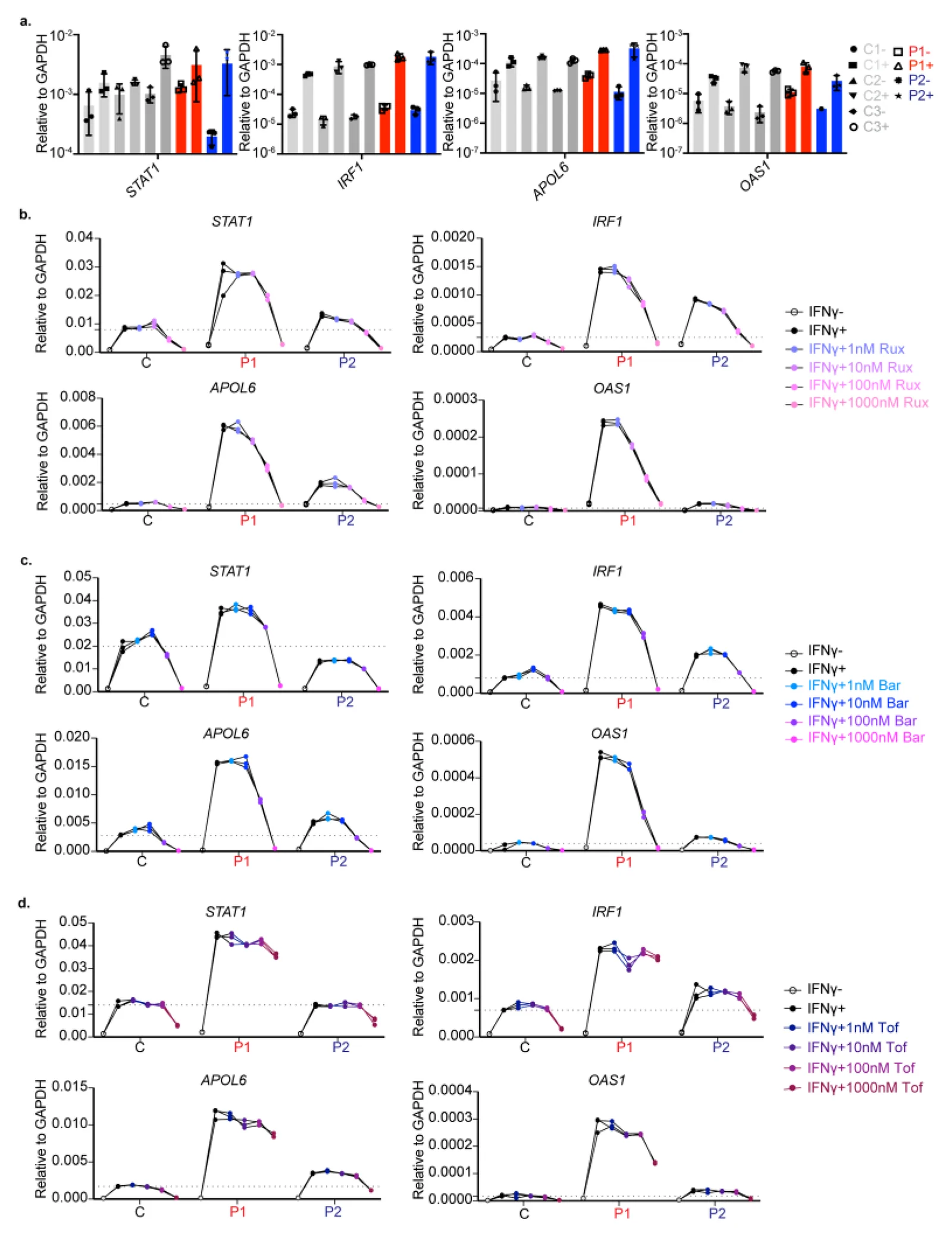
*STAT1* and its regulated gene expression following IFNγ stimulation with or without addition of JAK inhibitor. a) mRNA expression of *STAT1, IRF1, APOL6*, and *OAS1* in EPSC with or without 1h IFNγ stimulation; mRNA expression of *STAT1, IRF1, APOL6*, and *OAS1* in EPSC 24 hours following co-incubation with different concentrations of b) ruxolitinib (Rux), c) baricitinib (Bar) and d) tofacitinib (Tof).

In addition to p-STAT1 and t-STAT1 levels, we also noted a significantly increased expression of *STAT1* and its downstream genes (*IRF1, APOL6*, and *OAS1)* following IFNγ stimulation (Figure 6a). Similar to previous protein expression, we observed concordant changes of mRNA gene expression after co-incubation with specific JAKi as per previous, i.e. co-incubation with ruxolitinib and baricitinib resulted in marked reduction in the expression of *STAT1, IRF1, APOL6* and *OAS1;* which was not observed after co-incubation with tofacitinib (Figure 6b-d). In contrast, expression of non-IFN-regulated genes (*PEG3, NLRP4, and SOCS1, CD74*) was measured and did not exhibit similar changes following JAKi co-incubation (Figure E9).

### Corrected STAT1 phosphorylation and downstream gene expression in repaired STAT1-GoF EPSC

**Figure 7:**
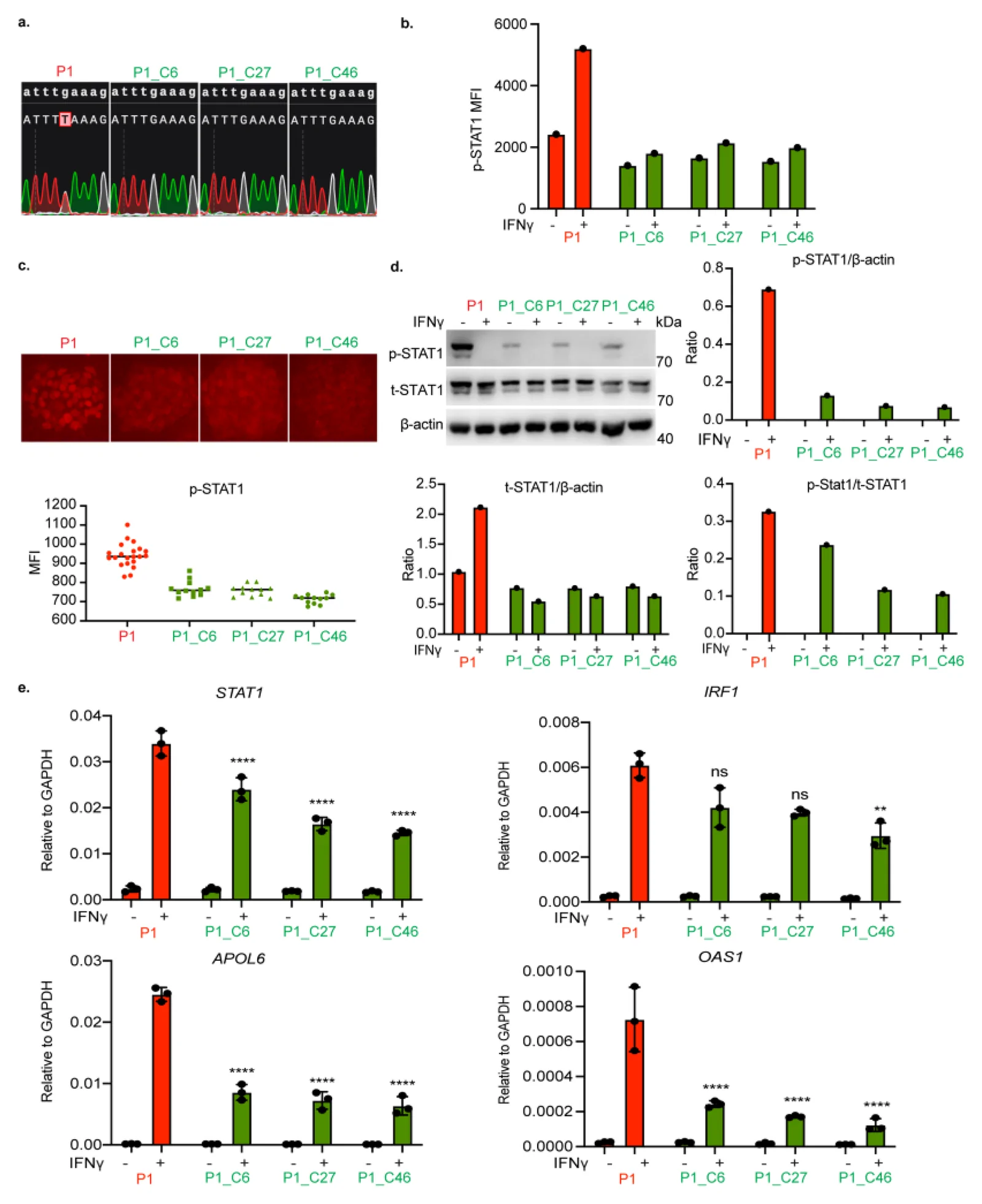
Repaired P1 EPSC demonstrating reduced STAT1 phosphorylation and downstream gene expression following IFNγ stimulation. a) Sanger sequencing confirming corrected GoF mutation; b) Flow cytometry of p-STAT1, c) Immunofluorescence staining of p-STAT1, d) Western blot of p-STAT1 and t-STAT1, e) qPCR quantification of *STAT1* and its downstream gene expression, in repaired and original P1 EPSC.

To further confirm the pathogenic function of the mutation in P1 (p.L358F) and evaluate the robustness of our EPSC platform, we repaired the *STAT1* GoF mutation on P1 ESPC by CRISPR/Cas9 editing. Three successfully repaired EPSC cell lines with 2 CRISPR/Cas9-blocking synonymous mutations (i.e. did not alter STAT1 protein sequence) were created and reconfirmed by Sanger sequencing (Figure 7a and Figure E10). We then examined the STAT1 phosphorylation levels of these repaired EPSC lines following IFNγ stimulation and compared them to the original P1-derived EPSC (rather than control EPSC, as p-STAT1 levels would not be directly comparable). All functional tests (Figure 7b-d) including flow cytometry, immunofluorescence staining and Western blot demonstrated significantly lower p-STAT1 expression in all 3 repaired EPSC cell lines compared to original P1-derived EPSC. Similarly, qPCR also demonstrated lower expression of both *STAT1* and its downstream genes following IFNγ stimulation (Figure 7e), with no obvious change in pluripotent and IFN receptor gene expression (Figure E11). All experiments were repeated two times to ensure consistent results.

## Discussion

We report the first proof-of-concept study on the feasibility of our patient-derived EPSC platform for modelling IEI. Despite being the most common inherited cause of CMC worldwide, STAT1-GoF research has remained limited, at least partly due to the lack of robust *ex vivo* models.^1^ Although a STAT1-GoF induced pluripotent stem cell line has been described, no functional tests or experiments to demonstrate disease modelling have been reported ^25^.

Using our EPC reprogramming approach, we were able successfully generate multiple and sustainable EPSC cell lines derived from different STAT1-GoF patients. Reprogramming EPSC from PBMC-derived EPC only requires simple venipuncture, which makes it more practical compared to other potential cell sources (such as fibroblasts - which would require more invasive patient biopsies). Functionally, these EPSC recapitulated patient-specific p-STAT1 abnormalities. Leveraging from established protocols for generation of various organoids from stem cell lines, we postulate that the generation of patient-derived organoids will further help study the differential effects of distinct STAT1 mutations upon specific organs or cells ^26–28^.

We also demonstrate the potential of our EPSC platform toward drug screening or personalized therapeutics. Although a variety of different JAKi have been reported be effective in STAT1-GoF, individuals will likely respond differently to specific JAKi (such as our experience with P1) ^11–17^. Co-incubation of P1-derived EPSC with either ruxolitinib and baricitinib (more JAK1/2-selective) led to marked improvement in STAT1 hyperactivation as well as downregulation of *STAT1* and its downstream genes, which was not seen with co-incubation with tofacitinib (more JAK1/3-selective). Interestingly, when measuring p/t-STAT1 as a fraction of peak STAT1, we did not observe a significant difference in rate of dephosphorylation between patients and control EPSC following only IFNγ withdraw (without JAKi). This suggests that STAT1 dephosphorylation may not be significantly different between STAT1-GoF patients and controls in the absence of JAKi, which has been previously described ^29^. Regardless, the significant difference in STAT1 hyperphosphorylation following co-incubation with specific JAKi recapitulated the real-life experience of P1 who responded to baricitinib but failed to respond to a more JAK1-selective agents. However, given the extreme heterogeneity of STAT1-GoF patients, individuals with different organ manifestations will likely respond differently to specific JAKi. Therefore, future prospective trials using this tailored approach to screen for therapies for different disease manifestations using patient-derived organoids would be of great interest. Such studies would also be invaluable to other IEI or rare diseases which generally lack disease-specific drugs. Even when drugs are available, therapeutic options for IEI are often limited or impose substantial patient risk (such as further immunosuppression or HSCT), making such *ex vivo* and patient-tailored platforms particularly appealing. For example, we postulate screening for potential JAKi for STAT3- and STAT6-GoF patients could be performed utilizing a similar approach ^30^.

Ultimately, the holy grail of treating inborn diseases is the complete cure of the underlying genetic defect – as recently exemplified by the examples of gene therapy for haemophilia ^31, 32^. Although a growing number of trials have been conducted, gene therapies for IEI are still not available for clinical use ^33^. Older IEI gene therapy trials were also associated with significant safety issues which put trial participants at risk developing leukaemia ^34–36^. Making use of CRISPR/Cas9 editing, we were able to successfully repair three different EPSC cell lines derived from our STAT1-GoF patient as evidenced by functional correction of p-STAT1 levels as well as reducing expression of *STAT1* and downstream genes. Experiments on *ex vivo* patient-derived EPSC allows us to study the effectiveness and functional correction of individualized gene therapy, as well as potential safety concerns (such as leukemogenesis) without imposing risk to patients. Further studies utilizing a customized gene editing approach would also enable us to study the existence of potential regulatory elements or interactions with other loci of interest.

As a proof-of-concept study, there are several limitations to this pilot study. First, EPSC were only reprogrammed from two STAT1-GoF patients and healthy controls. Given the vast heterogeneity among STAT1-GoF, it is possible that EPSC derived from different patients may exhibit different phenotypes or cellular characteristics. However, this also offers a unique opportunity to study and compare individual variants/mutations in the future. This potential utility will require larger sample sizes in future validation studies. Second, we were only able to conduct the drug screening using a limited number of JAKi, for example, we were unable to acquire upadacitinib (previously used by P1) for *ex vivo* testing. Further studies on primary patient cells were also limited due to difficulty in repeated patient blood sampling. However, this further highlights the clinical utility of our sustainable patient-derived EPSC cell lines, which can generate cells and organoids without needing for repeated patient sampling. Third, as a patient-derived cell line, we would not be able to discern potential contributions from undiscovered germline variations besides *STAT1*. However, this also offers the advantage of personalized disease modelling and may be more advantages in situations such as drug screening.

In conclusion, our proof-of-concept study demonstrates the potential of our patient-derived EPSC platform toward modelling IEI. Deriving EPSC from two STAT1-GoF patients, we were able to recapitulate functional abnormalities such as STAT1 hyperactivation. We propose this platform toward personalized disease modelling which can be used to selecting potential drug candidates, and ameliorates the need for repeated patient blood sampling. We were also able to repair known *STAT1* mutations by gene editing, in turn correcting p-STAT1 protein expression as well as the expression of *STAT1* and downstream genes. Other novel uses of this platform, such as comparing patient-derived EPSC vs. healthy EPSC with artificially engineered *STAT1* mutations, are currently underway. Validation studies with larger patient cohorts and different IEI from multi-center collaborations are also highly anticipated.

## Supplementary Material

Supplementary Materials

## Figures and Tables

**Table 1:** Clinical features of STAT1-GoF patients.

	Patient 1 (P1: g.29613G>T, c.1074G>T, p.Leu358Phe)	Patient 2 (P2: g.35561C>G, c.1398C>G, p.Ser466Arg)
Sex / Age (years)	Female / 21	Male / 28
Age of onset (years)	4	6
		
Infections		
- Bacteria	Acinetobacter baumannii, Campylobacter, Clostridioides difficile, Staphylococcus, Streptococcus, Neisseria	Haemophilus influenzae, Pseudomonas aeruginosa, Proteus, Staphylococcus,
- Mycobacteria		Mycobacterium avium complex, Mycobacterium kansasii
- Fungal	Chronic mucocutaneous candidiasis, P*neumocystis jirovecii, Talaromyces marneffei*	Chronic mucocutaneous candidiasis,
- Virus	Cytomegalovirus, Influenza, Swine flu	Cytomegalovirus, Herpes Simplex, Influenza
		
Autoimmunity	Autoimmune cytopenia, autoimmune hepatitis, inflammatory bowel disease	Autoimmune cytopenia, systemic lupus erythematosus
		
Other manifestations	Nil	Cerebral aneurysms
		
End organ damage	Bronchiectasis Bilateral sensorineural hearing loss Unilateral blindness	Bronchiectasis Unilateral sensorineural hearing loss
		
JAK inhibitors	Upadacitinib (Failure) Baricitinib (Responsive)	N/A

## Clinical Implications

Inborn errors of immunity, such as STAT1-Gain-of-Function, can be modelled using expanded potential stem cells reprogrammed from individual patients. This highlights the potential to personalize medicine for these rarer disorders.

## Capsule summary

Inborn errors of immunity often lack disease-specific models, limiting translational research and personalized medicine. Using STAT1-Gain-of-Funtion as an example, we demonstrate the potential of expanded potential stem cells to model patient-specific characteristics and personalize therapies.
