# Supplementary material for: Recapitulating and repairing STAT1 gain-of-function using patient-derived expanded potential stem cells (EPSC): a proof-of-concept of EPSC to study inborn errors of immunity
Source: J Allergy Clin Immunol. Author manuscript; Available in PMC 2026 Sep 4. (PMC7619439; doi:10.1016/j.jaci.2023.11.914)
Supplement: Supplementary file 1 [file EMS217777-supplement-Supplementary_Materials.docx]

**Table E1: List and sequence of oligonucleotides and primers used in this study.**

| Name | Description | Sequence |
| --- | --- | --- |
| *GAPDH* F | qPCR primer F | CCTCAACGACCACTTTGTCAAG |
| *GAPDH* R | qPCR primer R | TCTTCCTCTTGTGCTCTTGCTG |
| *NANOG* F | qPCR primer F | TGAACCTCAGCTACAAACAG |
| *NANOG* R | qPCR primer R | TGGTGGTAGGAAGAGTAAAG |
| *OCT4* F | qPCR primer F | CCTCACTTCACTGCACTGTA |
| *OCT4* R | qPCR primer R | CAGGTTTTCTTTCCCTAGCT |
| *SOX2* F | qPCR primer F | CCATGCAGGTTGACACCGTTG |
| *SOX2* R | qPCR primer R | TCGGCAGACTGATTCAAATAATACAG |
| *REX-1* F | qPCR primer F | AGAAACGGGCAAAGACAAGAC |
| *REX-1* R | qPCR primer R | GCTGACAGGTTCTATTTCCGC |
| *SALL4* F | qPCR primer F | GAAACCACATCCTTCCAGGCAC |
| *SALL4* R | qPCR primer R | GGATAAACGTGGAAGGGAGACTG |
| *STAT1* F | qPCR primer F | ATGGCAGTCTGGCGGCTGAATT |
| *STAT1* R | qPCR primer R | CCAAACCAGGCTGGCACAATTG |
| *IRF1* F | qPCR primer F | GAGGAGGTGAAAGACCAGAGCA |
| *IRF1* R | qPCR primer R | TAGCATCTCGGCTGGACTTCGA |
| *APOL6* F | qPCR primer F | CCACCTCTACTGCTGTCATCTC |
| *APOL6* R | qPCR primer R | GAGCGTTCCAACGTACCACTCA |
| *OAS1* F | qPCR primer F | AGGAAAGGTGCTTCCGAGGTAG |
| *OAS1* R | qPCR primer R | GGACTGAGGAAGACAACCAGGT |
| *IFNGR1* F | qPCR primer F | AGTGCTTAGCCTGGTATTCATCTG |
| *IFNGR1* R | qPCR primer R | GGCTGGTATGACGTGATGAGTG |
| *IFNGR2* F | qPCR primer F | CTCCATTCTGCCTGGGTGACAA |
| *IFNGR2* R | qPCR primer R | CGTGGAGGTATCAGCGATGTCA |
| *IFNAR1* F | qPCR primer F | CGCCTGTGATCCAGGATTATCC |
| *IFNAR1* R | qPCR primer R | TGGTGTGTGCTCTGGCTTTCAC |
| *IFNAR2* F | qPCR primer F | ACCGTCCTAGAAGGATTCAGCG |
| *IFNAR2* R | qPCR primer R | CCAACAATCTCAAACTCTGGTGG |
| *GATA2* F | qPCR primer F | GAACCGACCACTCATCAAGC |
| *GATA2* R | qPCR primer R | TTCTTCATGGTCAGTGGCCT |
| sgRNA1 | P1 GoF repair sgRNA | TTGCAATTTCACCAACAGTC |
| ssODN1 | P1 GoF repair ssODN  (lowercase show the edited nucleotides) | ATTTTTCTTTCCAGACTtTTaGTGAAATTGCAAGAGCTGAATTATAATTTgAAAGTCAAAGTCTTATTTGATAAGTAAGATATCTTTAACATTATATAAC |
| P1gDNA aF | P1 gDNA GoF amplify primer F | CTCAGTGGAGCAGAGGTACA |
| P1gDNA aR | P1 gDNA GoF amplify primer R | GAGACAAGAGTGAATCCCAC |
| P1gDNA sF | P1 gDNA GoF sequence primer F | CCCTCCATCTTTCCATGCTAC |
| P1gDNA sR | P1 gDNA GoF sequence primer R | AATGACCACAGAATCATGGC |
| sgRNA2 | T385M GoF installation sgRNA | GTCCACCAATGGCAGTCTGG |
| ssODN2 | T385M GoF installation ssODN | TTTAGGAAGTTCAACATTTTGGGCAtGCACACAAAAGTGATGAACATGGAGGAGTCCACCAATGGgAGcCTGGCGGCTGAATTTCGGCACCTGGTAGGGACATCAGTTTCCTCTCTATGT |
| T385M gDNA aF | T385M gDNA GoF amplify primer F | ACCAAGTAGCTGCTCAATAAAC |
| T385M gDNA aR | T385M gDNA GoF amplify primer R | CACAGAGGAGGAAAGATGGT |
| T385M gDNA sF | T385M gDNA GoF sequence primer F | GGTGCTGGTGCTTACATTTA |
| T385M gDNA sR | T385M gDNA GoF sequence primer R | GGAGAAAAGGTGGTAGAGTCAG |

Note: CRISPR/Cas9 edited sites are shown in lowercase

**Figure E1: Gene map of *STAT1* and loci of gain of function mutations.**

**Figure E2. Expansion of erythroblasts from PBMC from STAT1-GoF patients and controls.** a) Representative morphology of PBMC and PBMC-expanded EPC (magnification 200x); b)expression ofCD71 and CD135α by flow cytometry; c) downregulated expression of *CD19*, *CD3*, *CD14;* with upregulated expression of *CD71, HBA1, HBB,* and *HBG1.*

**Figure E3. Representative morphology of EPSC reprogrammed from PBMC-derived EPC from STAT1-GoF patients and controls.**

**Figure E4. Clearance of viral vector confirmed by a) RT-PCR and b) qRT-PCR.**

**Figure E5.** **Representativemorphology of patient and control EPSC during maintenance** at a) 100x, b) 400x and c) 400x magnification with Tra-1-60 staining.

**Figure E6: Expression of *STAT1*, IFN receptor genes and *GATA2* in patient and control EPSC.**

**Figure E7: p-STAT1, t-STAT1 and p/t-STAT1 ratioamong patient and control EPSC** following IFNγ deprivation within a) 1 and b) 8 hours.

**Figure E8: p-STAT1 expression in primary monocytes from STAT1-GoF patients’ peripheral blood mononuclear cells** a) at rest and after stimulation with IFNγ; b) following addition of ruxolitinib (Rux), c) baricitinib (Bar) and d) tofacitinib (Tof).

**Figure E9:** mRNA expression of non-IFN-regulated genesinEPSC with or without ruxolitinib following IFNγ stimulation.

**Figure E10: Sanger sequencing confirmation of CRISPR/Cas9 repaired P1 EPSC.** a) Sequencing results of candidates aligned to *STAT1* genome sequence. b) Sequencing peak maps of corrected colonies (P1_C6, P1_C27, P1_C46) with 2 Cas9-blocking synonymous mutations, and the original P1 EPSC which showed heterozygous GoF mutation.

**Figure E11: Expression of pluripotent and interferon receptor genes following IFNγ stimulation in original (red) and repaired (green) P1 EPSC.**

**Figure E1: Gene map of *STAT1* and loci of gain of function mutations.**

**Figure E2. Expansion of erythroblasts from PBMC from STAT1-GoF patients and controls.** a) Representative morphology of PBMC and PBMC-expanded EPC (magnification 200x); b)expression ofCD71 and CD135α by flow cytometry; c) downregulated expression of *CD19*, *CD3*, *CD14;* with upregulated expression of *CD71, HBA1, HBB,* and *HBG1.*

**Figure E3. Representative morphology of EPSC reprogrammed from PBMC-derived EPC from STAT1-GoF patients and controls.**

**Figure E4. Clearance of viral vector confirmed by a) RT-PCR and b) qRT-PCR.**

**Figure E5. Representative morphology of patient and control EPSC during maintenance** at a) 100x, b) 400x and c) 400x magnification with Tra-1-60 staining.

**Figure E6: Expression of *STAT1*, IFN receptor genes and *GATA2* in patient and control EPSC**

**Figure E7: p-STAT1, t-STAT1 and p/t-STAT1 ratioamong patient and control EPSC** following IFNγ deprivation within a) 1 and b) 8 hours.

**Figure E8: p-STAT1 expression in primary monocytes from STAT1-GoF patients’ peripheral blood mononuclear cells** a) at rest and after stimulation with IFNγ; b) following addition of ruxolitinib (Rux), c) baricitinib (Bar) and d) tofacitinib (Tof).

**Figure E9:** mRNA expression of non-IFN-regulated genesinEPSC with or without ruxolitinib following IFNγ stimulation.

**Figure E10: Sanger sequencing confirmation of CRISPR/Cas9 repaired P1 EPSC.** a) Sequencing results of candidates aligned to *STAT1* genome sequence. b) Sequencing peak maps of corrected colonies (P1_C6, P1_C27, P1_C46) with 2 Cas9-blocking synonymous mutations, and the original P1 EPSC which showed heterozygous GoF mutation.

**Figure E11: Expression of pluripotent and interferon receptor genes following IFNγ stimulation in original (red) and repaired (green) P1 EPSC.**
